# MADS-complexes regulate transcriptome dynamics during pollen maturation

**DOI:** 10.1186/gb-2007-8-11-r249

**Published:** 2007-11-22

**Authors:** Wim Verelst, David Twell, Stefan de Folter, Richard Immink, Heinz Saedler, Thomas Münster

**Affiliations:** 1 Department of Molecular Plant Genetics, Max-Planck-Institute for Plant Breeding Research, Carl-von-Linné-Weg, 50829 Cologne, Germany; 2 Department of Biology, University of Leicester, University Road, Leicester LE1 7RH, UK; 3 Business Unit Bioscience, Plant Research International, Droevendaalsesteeg 1, 6708 PB Wageningen, The Netherlands; 4 National Laboratory of Genomics for Biodiversity (Langebio), Centro de Investigación y de Estudios Avanzados (CINVESTAV-IPN), 36500 Irapuato, Guanajuato, Mexico

## Abstract

**Background:**

Differentiation processes are responsible for the diversity and functional specialization of the cell types that compose an organism. The outcome of these processes can be studied at molecular, physiologic, and biochemical levels by comparing different cell types, but the complexity and dynamics of the regulatory processes that specify the differentiation are largely unexplored.

**Results:**

Here we identified the pollen-specific MIKC* class of MADS-domain transcription factors as major regulators of transcriptome dynamics during male reproductive cell development in *Arabidopsis thaliana*. Pollen transcript profiling of mutants deficient in different MIKC* protein complexes revealed that they control a transcriptional switch that directs pollen maturation and that is essential for pollen competitive ability. We resolved the functional redundancy among the MIKC* proteins and uncovered part of the underlying network by identifying the non-MIKC* MADS-box genes *AGL18 *and *AGL29 *as downstream regulators of a subset of the MIKC* MADS-controlled genes.

**Conclusion:**

Our results provide a first, unique, and compelling insight into the complexity of a transcription factor network that directs cellular differentiation during pollen maturation, a process that is essential for male reproductive fitness in flowering plants.

## Abstract 2

Pollen transcript profiling of mutants defective in MADS-domain MIKC* protein complexes suggests they control a transcriptional network directing cellular differentiation during pollen maturation.

## Background

Cellular differentiation is the process responsible for the broad diversity of cell and tissue types that compose an organism. In plants this process directs the transcriptome of undifferentiated meristematic cells, which are essentially totipotent in nature, along one of many possible paths. The process and direction of cellular differentiation are guided both by intrinsic genetic and epigenetic factors and by external cues, such as hormones, that convey positional information to cells embedded within different organs or tissue types [1-5].

A few recent pioneering studies have provided valuable insights into the transcriptional signatures of differentiated cell types in plants [6-10]. These reports advance knowledge to the genomic level, representing a first, important step in elucidating the complexity of cellular differentiation, but they leave key questions unanswered. In particular, what is the sequence and dynamics of the regulatory processes that specify the path from inception to complete differentiation? This matter is extremely difficult to approach in higher plants, because most cell types are embedded within complex tissues, preventing the collection of purified cell types at discrete stages of their development. Birnbaum and colleagues [8] successfully analyzed the transcriptome of different cell types in roots by sorting cell-specific fluorescent marker lines. However, sampling of sequential developmental stages has been achieved in few reported studies. Kubo and coworkers [9] described transcriptional changes that occur during xylogenesis, and Honys and Twell [11] conducted transcript profiling on four stages of pollen development. Kubo and coworkers circumvented the problem of collecting developing xylem vessels by inducing the differentiation process *in vitro *[9], whereas Honys and Twell took advantage of the unique property of developing haploid male gametophytes (microspores and pollen grains) as the only differentiated plant cell types that remain physically isolated from neighboring cell types [11]. Moreover, pollen development proceeds along a stereotypical and unbranched pathway of differentiation, with well defined developmental stages, and is relatively synchronized within the anther, enabling the collection of homogenous cell populations at distinct stages of differentiation [11]. These features make the male gametophyte an attractive model for detailed analysis of the cell differentiation process in plants.

The high degree of specialization of mature male gametophytes manifests in the unique morphology, function, and transcriptome of pollen grains, which differ dramatically from all somatic cell types. Mature pollen grains are surrounded by a complex sculptured cell wall and contain three haploid cells [12]: a large vegetative cell that germinates to produce a rapidly growing pollen tube, and two sperm cells that are delivered within the pollen tube to the ovule. Developmental analysis revealed that the pollen transcriptome is uniquely adapted to its functions in gamete production and delivery, and is highly dynamic throughout development [11]. Throughout *Arabidopsis thaliana *(referred to hereafter as 'Arabidopsis') pollen development a total of nearly 14,000 genes are expressed, which corresponds to around 45% of the total transcriptome [11]. Although fully developed leaves and roots express at least 13,000 genes (around 60% of the genes represented on the ATH1 array [13]), mature pollen grains have a relatively small transcriptome of approximately 7,000 transcripts, with unusually large proportions of pollen-enriched (26%) and pollen-specific (11%) genes [7,11,13]. The availability of transcriptome and proteome reference datasets for Arabidopsis pollen [7,11,14-17] now enables a more systematic approach: functional characterization of regulatory networks that control pollen development.

Genetic screens in Arabidopsis have led to the identification of a number of interesting mutants with post-meiotic defects in pollen development (for review [18]). The various mutants disturb microspore polarity and cell division [19,20], cytokinesis [21], male germ cell division [22], pollen germination, tube growth, and guidance [23-25]. Although this approach has provided valuable information about key cellular processes in pollen development, the underlying regulatory pathways remain largely unknown. Pollen-expressed transcription factors are obvious candidates to play important regulatory functions. Even though more than 600 transcription factors are expressed throughout pollen development [11], very few have yet been functionally characterized. Although a forward genetics approach has led to the identification of DUO1 (a male germline-specific R2R3 MYB protein) as an essential regulator of sperm cell division and sperm cell formation [26], no information is currently available concerning the transcriptional networks that regulate cell differentiation and define cell-specific functions during pollen development.

Transcriptome analysis of wild-type (WT) pollen identified several transcription factor families that are under-represented whereas others are markedly over-represented in pollen [7,11]. Among the over-represented classes is the so-called 'AtMIKC*' subgroup of the MADS-box family: five of the six members of this subgroup (*AGL30*, *AGL65*, *AGL66*, *AGL94*, and *AGL104*) are predominantly expressed in pollen [7,27,28]. Although most AtMIKC* genes (with exception of *AGL65*) are already expressed at low levels during early pollen development, they are all maximally expressed during the last two developmental stages [11], and they are therefore expected to regulate transcription associated with pollen maturation [28]. AtMIKC* proteins interact with each other *in planta*, forming five heterodimeric transcription factor complexes that bind DNA with high specificity *in vitro*: AGL30/66, AGL65/66, AGL94/66, AGL30/104 and AGL65/104 [28]. Because various members of the MADS-box family regulate seed plant specific developmental programs [29,30], the AtMIKC* complexes are excellent candidates for regulators of the pollen maturation program.

Here, we aimed to elucidate the regulatory functions of the AtMIKC* complexes in pollen development. We studied pollen from *agl65*/*66*/*104 *triple mutant Arabidopsis plants, which functionally lack four of the AtMIKC* complexes (AGL65/66, AGL65/104, AGL30/66, and AGL94/66) while being markedly deficient in the fifth complex (AGL30/104). Even though we did not observe morphologic abnormalities in triple mutant pollen grains, we were able to show reduced pollen competitiveness *in vivo *and major changes in the pollen transcriptome. The absence of AtMIKC* complexes affected the expression of more than 1,300 genes during pollen maturation. Genes influencing a variety of functional processes (including major hormone pathways, metabolic processes, and various post-translational regulators) were affected. Our analyses show that AtMIKC* complexes act to repress immature pollen genes and activate mature pollen genes, thereby regulating a key transition during the pollen differentiation program. Moreover, we uncovered the extent of functional redundancy between the different AtMIKC* complexes by analyzing the pollen transcriptome of single and double AtMIKC* mutants. We then extended this analysis to reveal part of the underlying network, by identifying the *AGL18 *and *AGL29 *MADS-box genes as downstream regulators of a subset of the genes controlled by the AtMIKC* complexes. Our results provide unique insight into the complexity of a transcription factor network that directs differentiation during male reproductive cell development in plants.

## Results

### AtMIKC* mutant characterization

We previously described plants obtained from the Salk collection with a transferred DNA (T-DNA) insertion in the *AGL65*, *AGL66*, *AGL94 *and *AGL104 *genes, as well as three double mutant combinations of these insertion mutants [28]: *agl65/66*, *agl65/104*, and *agl66/104*. All plants were morphologically normal, but we observed specific *in vitro *pollen germination defects in double mutant combinations, indicating that the different AtMIKC* complexes are pair-wise functionally redundant. The most severe phenotype was observed for *agl66/104 *double mutant pollen, in which only low levels of two of the five complexes (namely AGL30/104 and AGL65/104) are present because of residual *AGL104 *expression [28]. Here, we describe a triple mutant (*agl65*/*66*/*104*), in whose pollen only the AGL30/104 complex is expected to be present, at low abundance. *In vitro *assays revealed that the germination of triple mutant pollen was almost completely blocked, similar to *agl66*/*104 *double mutant pollen [28]. The difference in *in vitro *germination between WT and triple mutant pollen is illustrated in Additional data file 1 (panel a).

In order to obtain initial clues about the biologic function of the AtMIKC* complexes, we examined the morphology of triple mutant pollen grains. We examined pollen nuclei after 4',6-diamidino-2-phenylindole (DAPI) staining and sectioned pollen grains that were histochemically stained for callose, cellulose, and pectin, but we observed no differences between triple mutant and WT pollen grains (Additional data file 1 [panels d and g to j]). Pollen viability (Additional data file 1 [panel c]) and dimensions (data not shown) were also unchanged. In addition, scanning and transmission electron microscopy did not reveal obvious differences in pollen surface and ultrastructure (Additional data file 1 [panels e and f]). Mutant pollen grains also appeared to be properly dehydrated at anthesis when tested with the water soluble dye 8-hydroxypyrene-1,3,6-trisulfonic acid (HPTS) [31] (data not shown). We thus concluded that triple mutant pollen is morphologically indistinguishable from WT pollen.

Subsequently, we performed pollination assays to investigate the *in vivo *performance of *agl65*/*66*/*104 *triple mutant pollen. Triple mutant pollen grains germinated efficiently on WT pistils and pollen tube growth through the style was comparable to that of WT tubes (Additional data file 1 [panel b]). These results suggest that the *in vivo *performance of triple mutant pollen is not impaired, which is in agreement with the normal seed set of triple mutant plants (53 ± 3 seeds per silique versus 55 ± 2 in WT; *n *= 15). This discrepancy between pollen germination *in vivo *and *in vitro *urged us to investigate whether the AtMIKC* mutant alleles are efficiently transmitted compared with the WT alleles.

Reciprocal test-crosses allowed us to assess directly the relative competitiveness of single, double, and triple mutant pollen (Table 1). In these experiments we reciprocally crossed WT plants with plants homozygous for one or two AtMIKC* mutations, and heterozygous for another AtMIKC* mutation. Testing the transmission of the allele segregating in one of the parents of such a cross allowed us to compare directly the relative competitive *in vivo *performance of gametes carrying a mutation in an AtMIKC* gene in a predefined mutant background with the performance of gametes lacking that mutant allele. This approach revealed that *agl65*/*66 *double mutant pollen was slightly less competitive than *agl65 *pollen. In addition, although *agl65*/*104 *pollen was less competitive than *agl104 *pollen, *agl66*/*104 *double mutant pollen was even much less competitive, relative to *agl104*. We also found that *agl65*/*66*/*104 *triple mutant pollen was far less competitive than *agl65*/*104 *double mutant pollen. Step-wise removal of functional AtMIKC* genes thus enhanced the *in vivo *phenotype in a manner comparable to that observed in *in vitro *assays (described by Verelst and coworkers [28]). We conclude that pollen grains deficient for multiple AtMIKC* complexes have normal morphology, but that mutant pollen tubes are less competitive than WT, revealing an important post-germination role for these regulators *in planta*.

**Table 1:** AtMIKC* mutations decrease competitive ability of pollen

Female	Male	*n*	Allele	TE	χ^2^
Wild-type Col-0	*agl65*/*agl66*/*AGL66*	64	*agl66*	68	2.25
*agl65*/*agl66*/*AGL66*	Wild-type Col-0	ND	*agl66*	ND	
Wild-type Col-0	*agl104*/*agl65*/*AGL65*	61	*agl65*	65	2.77*
*agl104*/*agl65*/*AGL65*	Wild-type Col-0	29	*agl65*	93	0.03
Wild-type Col-0	*agl104*/*agl66*/*AGL66*	40	*agl66*	33	10.00**
*agl104*/*agl66*/*AGL66*	Wild-type Col-0	ND	*agl66*	ND	
Wild-type Col-0	*agl65*/*agl104*/*agl66*/*AGL66*	66	*agl66*	29	19.64**
*agl65*/*agl104*/*agl66*/*AGL66*	Wild-type Col-0	58	*agl66*	107	0.07

Reciprocal testcrosses were performed with plants homozygous for one or two AtMIKC* mutations, and heterozygous for another AtMIKC* mutation. The total number of progeny analyzed (*n*) and transmission efficiency (TE; expressed as percentage, as defined by Howden and coworkers [59]) of the segregating mutant allele relative to that of the wild-type allele are presented, together with the χ^2 ^value for transmission in each cross. Significant differences are identified between transmission of mutant and wild-type alleles at *α = 0.10 and **α = 0.01. ND, not done.

### The transcriptome of *agl65/66/104 *triple mutant pollen

In order to identify downstream target genes of the AtMIKC* transcription factor complexes, we used transcript profiling on *agl65*/*66*/*104 *triple mutant pollen. We harvested mature pollen grains (in triplicate) from open flowers of WT and homozygous triple mutant plants, isolated RNA, and performed microarray hybridizations using the 23 k whole-genome chip (ATH1; Affymetrix Inc., Santa Clara, CA, USA). We first tested the reliability of our dataset by verifying that our samples had not been contaminated with RNA from sporophytic tissues. None of numerous genes specifically and highly expressed in other floral organs (including genes encoding abundant photosynthetic proteins) were detected, indicating the purity of our pollen RNA samples (Additional data file 2).

We analyzed triple mutant and WT pollen datasets using the Cyber-T statistical program [32], using a posterior probability of differential expression (PPDE) of 0.95 as cut-off. This implied that there was at least a 95% chance that the selected genes were not false positives but were truly differentially expressed between the genotypes, and this threshold corresponded to log-transformed *P *values (Bayeslnp) of 0.0008 or less (see Materials and methods, below). We found that 1,353 genes were significantly and reproducibly affected in mature pollen, 804 of which differed by more than twofold. Compared with WT pollen, 606 genes were downregulated and 747 genes were upregulated in the triple mutant. The complete dataset is presented in Additional data file 2.

**Figure 1:**
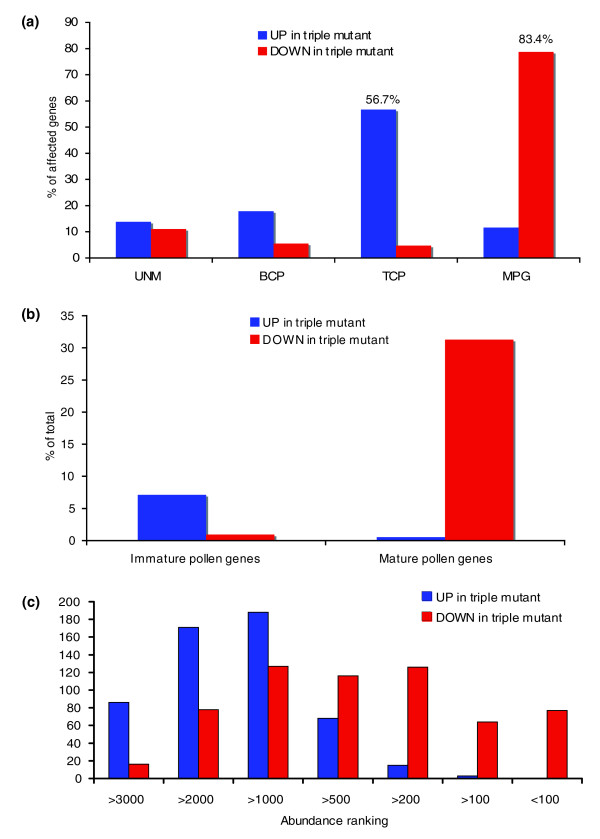
AtMIKC* complexes regulate a transcriptional switch during pollen maturation. **(a) **Of the genes downregulated in *agl65*/*66*/*104 *triple mutant pollen, 83.4% exhibit peak expression at the mature pollen grain (MPG) stage of wild-type (WT) pollen development (according to Honys and Twell [11]), whereas 56.7% of the genes upregulated in this mutant peak during the immature tricellular stage (tricellular pollen [TCP]). In total, 83.5% of the upregulated genes peak during the three immature stages (unicellular microspores [UNM], BCP, and TCP). **(b) **The AtMIKC* complexes contribute quite significantly to the transcriptional changes that occur during pollen maturation. **(c) **We ranked all genes that were consistently called present in WT pollen according to their expression level in mature WT pollen, in descending order (the highest expressed gene received number 1). In this graph the ranking numbers of all genes upregulated and downregulated in triple mutant pollen are plotted, revealing that AtMIKC* complexes predominantly activate high-abundance and medium-abundance transcripts, while repressing low-abundance and medium-abundance transcripts. All calculations related to these graphs are included in Additional data file 2.

Subsequently, we compared our results with the reference dataset of Honys and Twell [11], who characterized the transcriptome of four stages of pollen development: unicellular microspores (UNM), bicellular pollen (BCP), tricellular pollen (TCP), and mature pollen grains (MPG). We found that 83.4% of all genes downregulated in triple mutant pollen are expressed maximally during the MPG stage in WT pollen. On the other hand, 83.5% of the upregulated genes exhibit peak expression during the immature stages of WT pollen development (Figure 1a). These observations show that many mature pollen (MP) genes are only partially induced during triple mutant pollen development, whereas numerous immature pollen (IP) genes are incompletely repressed.

We then estimated the contribution of the AtMIKC* complexes to all transcriptional changes that occur during pollen maturation. We compiled a list of all IP and MP genes in Arabidopsis pollen, again making use of the reference dataset of Honys and Twell [11]. Genes were classified as IP if their expression in mature pollen decreased by at least 50% relative to the TCP stage. Similarly, we termed genes MP if their WT expression increased at least 50% in mature pollen, relative to the three immature stages. Of the 3,972 IP genes we identified with these stringent criteria, 283 (7.1%) were reliably upregulated in the triple mutant, and only nine were downregulated. Of the 959 MP genes, 300 (31.3%) were downregulated and 21 were upregulated (Figure 1b). Therefore, a large portion of the transcriptional changes that occur during pollen maturation depends on the AtMIKC* complexes.

To verify whether low-abundance and high-abundance transcripts were equally represented among the genes affected in triple mutant pollen, we compiled a list of all genes consistently called present in our three WT replicate samples (3,819 in total), and we then ranked them in descending order. In each individual WT sample between 4,250 and 5,100 genes were called present, but the overlap was only 3,819. Under our conditions we routinely scored between 4,250 and 6,000 present calls in pollen, although other studies reported higher numbers of genes expressed in pollen [7,11]. We then examined the ranking numbers of all genes that were upregulated and downregulated in triple mutant pollen. We noticed that the genes that were downregulated in triple mutant pollen mostly belonged to high-abundance and medium-abundance classes, whereas the genes upregulated in triple mutant pollen predominantly belonged to medium-abundance and low-abundance classes (above rank 1,000; Figure 1c). In particular, of the 200 most abundant transcripts in mature pollen, 141 were reproducibly downregulated in triple mutant pollen. Therefore, the AtMIKC* complexes mainly induce abundant transcripts in mature pollen, and repress transcripts of moderate or low abundance. This further highlights the important role played by the AtMIKC* complexes in shaping the mature pollen transcriptome. We conclude that the AtMIKC* complexes play a major role in regulating the transcriptional switch during pollen maturation. In addition, they appear to be required for the repression of certain sporophytic transcripts in mature pollen, as illustrated in Additional data file 2.

### Function of putative AtMIKC* target genes

The genes controlled by the AtMIKC* complexes are related to a wide variety of biologic processes, which indicates that multiple pathways are affected. An overview of the major functional classes of AtMIKC*-controlled genes is presented in Additional data file 2. An example of an entire pathway regulated by the AtMIKC* complexes is cell wall component synthesis. Analysis with MapMan software [33] revealed that the biosynthesis genes for uridinediphosphate (UDP)-xylose, UDP-arabinose, UDP-rhamnose, UDP-fucose, UDP-fructose, and UDP-glucose (all IP-specific processes) were upregulated in triple mutant pollen, whereas genes for UDP-galacturonic acid synthesis (which are MP specific) were downregulated (Additional data file 3).

An important class of AtMIKC*-regulated genes is related to hormone metabolism and signaling. Our experiments suggested that the AtMIKC* complexes may repress the auxin signaling pathway in maturing pollen (more precisely the auxin receptor TIR1 [34]), and activate the cytokinin and ethylene signaling pathways, as well as the synthesis of abscisic acid and methyl jasmonate (Additional data file 2, and confirmations by reverse transcription polymerase chain reaction [RT-PCR] in Additional data file 5). The observation that jasmonic acid is required for anther dehiscence and correct pollen maturation [35] is highly relevant in this context. It would be interesting to investigate the contribution of each of these hormonal pathways to the *agl65*/*66*/*104 *triple mutant phenotype, in order to uncover the role played by hormones in pollen development and function, a topic that has thus far remained largely unstudied.

Another intriguing observation was the upregulation of *PHYB *(which encodes phytochrome B) in triple mutant pollen, together with the entire morning component of the central circadian clock oscillator: *CCA1*, *LHY*, and *PRR7 *(Additional data file 2) [36,37]. Moreover, *ZTL *(*ZEITLUPE*) and *FKF1 *(*FLAVIN-BINDING KELCH DOMAIN F-BOX PROTEIN*), which encode F-box proteins with important functions in the circadian clock, were also upregulated. The dataset of Honys and Twell [11] indicated that these genes were most strongly expressed in IP stages, implying that the AtMIKC* complexes normally repress them during pollen maturation. We confirmed the differential expression of *CCA1 *and *ZTL *by RT-PCR (Additional data file 5). Intriguingly, the expression level of these genes was consistently elevated in triple mutant pollen throughout the day, but no difference could be observed in their mRNA levels between morning and evening, in neither WT nor triple mutant pollen (Verelst W, Münster T, unpublished data). This observation strongly suggests a lack of conventional circadian rhythms in pollen. The function of CCA1 and the other morning components of the clock could conceivably be related to red light signaling rather than to a circadian rhythm. Again, these processes remain entirely unstudied in pollen.

### Functional redundancy between the AtMIKC* complexes

**Figure 2:**
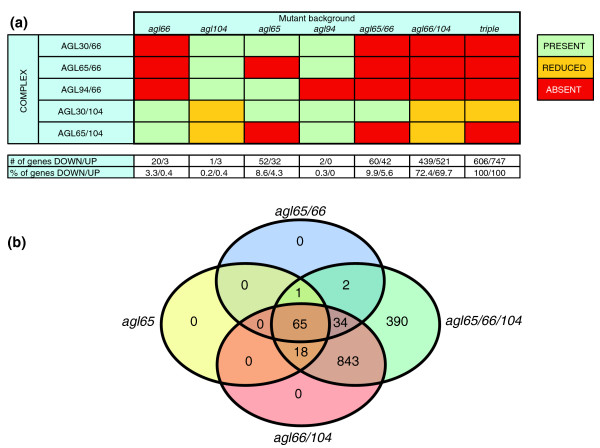
Functional redundancy is high among AtMIKC* complexes. **(a) **Overview of the presence (green), absence (red), and reduced abundance (orange) of the five MIKC* complexes in different mutant backgrounds. Values represent the number (upper row) and percentage (lower row) of AtMIKC*-controlled genes that were downregulated and upregulated in each of the mutants. **(b) **Graphical representation of the numbers of significantly affected genes shared by different mutants (based on the FIRe macro [38]); virtually all genes affected in *agl65 *and *agl65/66 *pollen are also affected in *agl66*/*104 *pollen.

The AtMIKC* proteins in Arabidopsis pollen bind to DNA as five heterodimeric complexes. *In vitro *pollen germination assays suggested redundancy between the two AGL65 complexes (AGL65/66 and AGL65/104), and also between the two AGL30 complexes (AGL30/66 and AGL30/104). The AGL66 and AGL104 proteins thus appeared to be functionally interchangeable, whereas AGL30 and AGL65 were not [28]. Our present analysis of the *agl65*/*66*/*104 *triple mutant pollen transcriptome provided us with a list of genes whose expression depends on the AtMIKC* complexes. However, because all five complexes were affected in this mutant background, it did not allow us to assess the contribution made by the individual AtMIKC* complexes. To address this issue, we compared the *agl65*/*66*/*104 *triple mutant pollen transcriptome with that of various single and double AtMIKC* mutants, in which different combinations of AtMIKC* complexes were either absent or strongly reduced in abundance [28]. We thus investigated the pollen transcriptome of the *agl66*, *agl104*, *agl65*, *agl94*, *agl65/66*, and *agl66/104 *mutants, each in duplicate, and compared the expression level of the 1,353 AtMIKC*-regulated genes with that in triple mutant and WT pollen. Again, these datasets were entirely free from sporophytic contaminants (Additional data file 2). Figure 2a illustrates the presence and absence of the five AtMIKC* complexes in each of these mutants.

Because the different mutants were analyzed in four independent experiments, we allowed a false-positive rate of up to 10% (PPDE > 0.90 in the Cyber-T analysis), relative to the WT control samples (see Materials and methods, below). In this way we minimized the potential influence of slight environmental differences on the expression of AtMIKC*-controlled genes in the different experiments. For each gene we calculated the expression level in each mutant, relative to the corresponding WT control. All genes reliably affected in the single and double mutants were also affected in the triple mutant. This enabled us to express the transcriptional changes in each single and double mutant as a percentage of the total number of AtMIKC*-regulated genes we had identified in our analysis of the triple mutant (Figure 2a).

The complete redundancy between the AGL66 and AGL104 proteins was convincingly confirmed. Loss of the AGL30/66, AGL65/66, and AGL94/66 complexes (in *agl66 *mutant pollen, indicated by red boxes) had barely any impact on the pollen transcriptome (Figure 2a), implying that the two remaining complexes (AGL30/104 and AGL65/104) were sufficient to regulate the expression of virtually all AtMIKC*-dependent genes. Similarly, the strongly reduced abundance of AGL30/104 and AGL65/104 in *agl104 *mutant pollen (indicated by orange boxes) affected fewer than 0.5% of AtMIKC*-controlled genes. Hence, AGL65/66 and AGL65/104 form a functionally redundant pair, and the same is true for AGL30/66 and AGL30/104. The contribution of the two AGL65 complexes to the overall AtMIKC* function could be estimated from analysis of the *agl65 *mutant, in which both AGL65/66 and AGL65/104 were dysfunctional. In the *agl65 *single mutant, 8.6% of the genes activated by the AtMIKC* complexes and 4.3% of all repressed genes were significantly affected. AGL65 complexes together thus regulate only a small subset of the AtMIKC*-dependent genes. The presence (at normal levels) of only the AGL30/104 complex was sufficient to ensure the correct regulation of the vast majority of AtMIKC*-regulated genes; only 9.9% of the activated genes and 5.6% of the repressed genes were affected in the *agl65*/*66 *mutant.

Functional redundancy is thus very high among the AtMIKC* complexes. This was further clarified by our analysis of the *agl66*/*104 *double mutant, which has reduced levels of AGL30/104 and AGL65/104 complexes, and lacks the other three AtMIKC* complexes. In this background, the expression of around 70% of all genes affected in the triple mutant was significantly changed. Because the only relevant difference from the *agl65*/*66 *mutant is the reduced level of AGL30/104 complex, this highlights the important role played by this particular complex in the AtMIKC* network. The overlap between different AtMIKC* mutants is graphically displayed in Figure 2b (based on the FIRe macro [38]). It illustrates that virtually all genes affected in *agl65 *and *agl65/66 *mutant pollen are also affected in *agl66*/*104 *pollen.

In further analyses we also took into account the extent of changes in expression level in each single and double mutant (Additional data files 2 and 4). This approach revealed how each individual AtMIKC* complex contributes to the expression of each AtMIKC*-controlled gene. We found that only 21 genes are specifically regulated by AGL65 complexes, whereas 657 genes depend exclusively on AGL30 complexes for their proper expression. For 60 genes the analysis suggested additive regulation by both AGL30 and AGL65 complexes, whereas 218 genes behave as redundantly regulated by all AtMIKC* complexes (Additional data files 2 and 4). Although the contribution of the AGL94/66 complex remained unresolved after these experiments, the limited changes in the pollen transcriptome of the *agl94 *mutant (Figure 2a) suggested either that this complex regulates the expression of very few genes or that it acts entirely redundantly with the other AtMIKC* complexes. Moreover, previous experiments suggested that *AGL94 *might even be a recent pseudo-gene; the AGL94/66 complex could not be demonstrated *in planta*, DNA binding was suboptimal *in vitro*, and *AGL94 *transcript levels are very low in mature pollen [28].

### AtMIKC* complexes control a MADS network in pollen

Among the putative targets of AtMIKC* complexes in pollen, we identified four MADS box genes: *AGL30*, *AGL65*, *AGL18*, and *AGL29*. The expression of *AGL30*, *AGL18*, and *AGL29 *was affected to a comparable extent in *agl66*/*104 *and triple mutant pollen, and not in any other mutant we examined. As explained in Additional data file 4, this indicated that they are specifically regulated by the AGL30 complexes (*AGL30 *and *AGL18 *are shown as examples in Additional data file 4). *AGL30 *and *AGL65 *expression was induced, and *AGL18 *and *AGL29 *expression strongly reduced in triple mutant compared with WT pollen, which we confirmed by RT-PCR for the latter two cases (Additional data file 5). The AtMIKC* complexes thus repress *AGL30 *and *AGL65 *expression in a negative feedback loop, and activate the other two MADS-box genes during WT pollen development. *AGL18 *and *AGL29 *are the only two non-MIKC* MADS box genes expressed at high levels in pollen, and neither has thus far been functionally characterized. *AGL18 *belongs to the MIKC^c ^MADS subgroup, whereas *AGL29 *is a so-called 'type I' MADS box gene of the Mα clade [39].

To investigate further this MADS network, we evaluated the genetic interactions between the different MADS proteins in pollen by studying the transcriptomes of *agl18 *and *agl29 *single mutant pollen. The T-DNA insertion line we used for *AGL18 *was previously named *agl18-2 *[40]. Because all genes affected in the *agl18-2 *and *agl29 *mutants were also affected in *agl65*/*66*/*104 *triple mutant pollen, we concluded that both MADS proteins regulate the expression of a subset of AtMIKC*-controlled genes; AGL18 repressed around 2% of the AtMIKC*-controlled genes and AGL29 repressed about 7% (Additional data file 2). These experiments also showed that *AGL29 *expression was fourfold upregulated in *agl18-2 *mutant pollen, revealing AGL18 to be a strong repressor of *AGL29 *transcription. The expression level of *AGL29 *is thus a dynamic balance between its activation by the AGL30 complexes and repression by AGL18, which in turn is also activated by the AGL30 complexes. Conversely, AGL29 does not regulate AGL18 because *AGL18 *expression levels were unchanged in *agl29 *mutant pollen.

The main function of AGL18 in pollen appears to be modulation of *AGL29 *expression. Only 24 other genes were upregulated, and four genes (apart from *AGL18 *itself) were downregulated in *agl18-2 *mutant pollen. In *agl29 *mutant pollen, 76 genes were upregulated and 17 genes were downregulated (apart from *AGL29 *itself). Overall, the genes regulated by AGL29 were more strongly affected in *agl65*/*66*/*104 *than in *agl2*9 mutant pollen, suggesting that AGL29 is not the only factor regulating their expression. In addition, even though the functional loss of AGL18 in *agl18-2 *mutant pollen strongly increased *AGL29 *mRNA abundance, only six of the 93 AGL29-dependent genes were affected in *agl18-2 *pollen. This could indicate that AGL29 protein abundance and/or activity is further modulated at the post-translational level.

### Architecture of the network downstream of the AtMIKC* complexes

We recently reported that the Arabidopsis MIKC* transcription factor complexes preferentially bind so-called myocyte enhancer factor (MEF)2-type CArG-box motifs *in vitro*, which are enriched in the proximal promoters of late pollen-specific genes (consensus CTA(A/T)_4_TAG, CTA(T)_3_TAG and CTA(A)_3_TAG [28]). Here we used this information to estimate the number of potential direct targets genes of the AtMIKC* complexes in pollen.

We screened the 3000 bp promoters, 5'-untranslated regions, and first and second introns of all AtMIKC*-controlled genes identified in this study for the presence of AtMIKC*-binding motifs, and found them in 320 out of the 1,353 genes (Additional data file 2). These genes are potential direct targets of the AtMIKC* complexes. Of the 179 TCP/MPG-specific genes with AtMIKC* binding sites in their upstream sequence we previously identified *in silico *[28], 80 (45%) were significantly affected in triple mutant pollen (Additional data file 2), indicating that the binding sites we determined *in vitro *are also relevant *in vivo*. Of at least 45 non-MIKC* transcription factors that were among the affected genes (Additional data file 2), four were classified as potential direct target genes: *WRKY34*, *MYB97*, *EIL1 *(*ETHYLENE-INSENSITIVE3-LIKE 1*), and bZIP protein encoding gene At5g49450. Interestingly, *WRKY34 *was one of 34 AtMIKC*-controlled genes that contained two or more MEF2-motifs in their upstream and/or intronic region (Additional data file 2). Following the floral quartet model [41,42], the presence of two CArG-like motifs in a regulatory sequence could suggest that MADS proteins bind these sites as a tetrameric 'higher order complex', which bends DNA by binding to two distinct CArG motifs.

To investigate whether the AtMIKC* proteins in pollen could function as higher order complexes we tested their interactions in a yeast-three-hybrid experiment. We found that the AGL66 and AGL104 proteins, which were unable to interact directly [28], could interact with themselves and with each other in yeast when AGL30 or AGL65 was present as a bridge (Additional data file 6). Also, AGL30 and AGL65 could interact with themselves and with each other in the presence of AGL66 or AGL104. Taking into account the quartet model, these observations suggest that AGL30, AGL65, AGL66, and AGL104 are able to interact together to form tetrameric complexes, in which at least one protein can be present as a homodimer. AGL18 and AGL29, on the other hand, appear to function in independent complexes and not as part of AtMIKC* higher order complexes, as indicated by their lack of detectable interaction with the AtMIKC* complexes (Additional data file 6).

## Discussion

**Figure 3:**
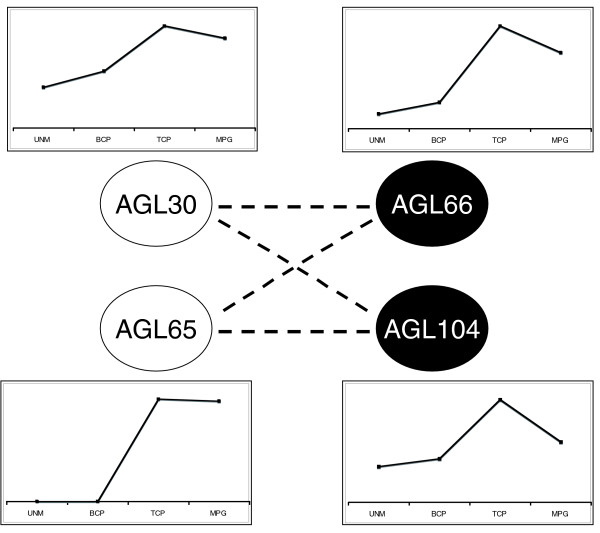
Evolution of the AtMIKC* network. After duplication of one ancestral *AGL30*-like gene and one ancestral *AGL66*-like gene, the paralogs AGL30 and AGL65 maintained the ability to interact with AGL66 and AGL104, resulting in the complex AtMIKC* network that exists in pollen of extant Arabidopsis. Although *AGL30*, *AGL66*, and *AGL104 *expression is initiated early, starting from the unicellular microspore stage, *AGL65 *is only activated later, during the tricellular stage. This change in expression profile may have been responsible for the apparent loss of control over numerous genes by the AGL65 complexes (which was suggested by our analysis in Additional data file 4). BCP, bicellular pollen; MPG, mature pollen grains; TCP, tricellular pollen; UNM, unicellular microspores.

From an evolutionary point of view, our data clearly illustrate the functional divergence that occurs within a transcription factor network. Moreover, they provide a good example of 'global conservation' after gene duplication, as defined by Veron and coworkers [43]. The ancestral scenario most likely featured a single heterodimeric (and/or higher order) MIKC* complex [28], with AGL66 and AGL104 proteins on the one hand, and AGL30 and AGL65 on the other, later arising from single gene duplication events [44]. The former duplication probably occurred much more recently than the latter one [28]. Throughout evolution these paralogous pairs retained their interaction partners and the ability to function in specific heterodimeric and higher order complexes (Figure 3 and Additional data file 6) [28]. Although the more recently derived paralogs AGL66 and AGL104 are still functionally interchangeable, as shown by transcriptome profiling of the respective single mutants (Figure 2a), we observed a pronounced functional difference between the older paralogous pair, AGL30 and AGL65. We can assume that both proteins were initially identical and functionally interchangeable, but the AGL65 complexes in extant Arabidopsis appear to regulate only few genes independently from the AGL30 complexes (category 1 in Additional data file 4). The latter, on the other hand, appear to control at least 657 genes on which the AGL65 complexes have no impact, whereas the functional overlap between AGL30 and AGL65 remains high (categories 2 to 5 in Additional data file 4).

These observations suggest that AGL30 could have retained the ancestral AtMIKC* function, whereas its paralog AGL65 has lost control over a considerable number of the initial AtMIKC*-regulated genes. An obvious difference between AGL30 and AGL65 lies in their expression profiles during pollen development. Like *AGL66 *and *AGL104*, *AGL30 *is expressed as early as the UNM stage, but *AGL65 *only appears to be activated during the TCP stage (Figure 3). It is therefore possible that the AGL30 complexes already initiate an important part of their regulatory function during the UNM and/or BCP stages, when AGL65 is absent. In this scenario a promoter mutation may thus have been the direct cause for the reduced importance of AGL65 in the AtMIKC* network. It would therefore be interesting to investigate how the MIKC* network has evolved in pollen from other angiosperm lineages, in which evolution may have taken a different path.

It is intriguing that even though more than 1,300 transcripts are misregulated in our triple mutant pollen, the basic cellular organization and characteristic properties of mature pollen (such as desiccation, morphology, and the ability to germinate and fertilize ovules *in vivo*) appear to be largely unaffected in *agl65*/*66*/*104 *triple mutant pollen. The reason for these incomplete functional defects (Table 1 and Additional data file 1) is most likely the 'leakiness' of the *agl104 *allele we used to construct our double and triple mutants (SALK_098698). The T-DNA insertion was located in intron 5, and we could detect up to 30% of the WT *AGL104 *transcript level in this mutant [28]. This implies that the AGL30/104 complex is still present in our *agl65*/*66*/*104 *triple mutant. In our analyses we clearly identified this complex (most likely together with its fully redundant counterpart AGL30/66) as the crucial component of the AtMIKC* network, being capable of correctly regulating over 90% of all identified AtMIKC*-controlled genes without assistance of the other complexes (in the *agl65*/*66 *double mutant; Figure 2a). Reduction in AGL30/104 complex abundance (by introducing the weak *agl104 *allele into an *agl65*/*66 *background) had a large impact on the pollen transcriptome (Figure 2). For 133 out of the 218 genes we identified as redundantly regulated by all AtMIKC* complexes, our analysis indicated that they require normal levels of at least one AtMIKC* complex for their proper expression, whereas the remaining 85 genes require lower levels (categories 4 and 5 in Additional data file 4). This observation illustrates that the threshold for AtMIKC* complex levels differs between the various genes they regulate. It is therefore easy to envision that the strongly reduced (but not negligible) abundance of this important complex in our triple mutant could have masked many more AtMIKC*-controlled genes. Even though we found AtMIKC* complexes to regulate a significant percentage of the transcriptional changes that occur during pollen maturation (about 30%; Figure 1b), we probably still severely underestimated their functional importance. Further reduction of AGL30/104 complex abundance might affect additional genes, which require only very low levels of AGL30/104 for their proper regulation.

Additional evidence for this assumption was provided by an alternative allele of *agl104 *(SALK_066443). In this second allele a T-DNA insertion was also located in the fifth intron, but *AGL104 *expression was considerably lower than in SALK_098698 (data not shown). Pollen transmission efficiency was only around 5% for this stronger *agl104 *allele when combined with *agl66 *(data not shown), as compared with 29% for the weaker *agl104 *allele (Table 1), and in spite of exhaustive efforts we were unable to combine this stronger allele into double mutants with *agl65 *or *agl66*. This indicates that further reduction in AGL30/104 more severely impairs pollen function. Although the weaker *agl104 *allele provided a unique opportunity to study the role of this transcription factor network in pollen, it also masked some important aspects of AtMIKC* complex function. This illustrates the balance that must be found when studying essential developmental regulators, and underlines the pronounced quantitative effects that the AtMIKC* complexes have on the expression of the genes they regulate. Although other, AtMIKC*-independent regulatory networks are probably involved as well, our experiments strongly indicate that the AtMIKC* network plays a major role in regulating pollen maturation and reproductive fitness.

## Conclusion

**Figure 4:**
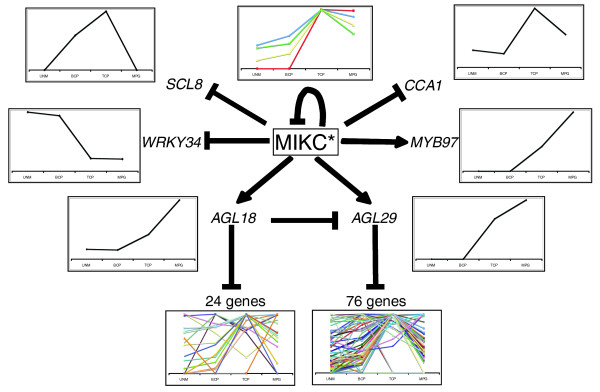
The sequential action of mutually interacting transcription factors directs transcriptome dynamics during pollen maturation. AtMIKC* complexes repress immature pollen specific transcription factors such as WRKY34, and activate mature pollen specific transcription factors such as AGL18 and AGL29. These in turn predominantly repress transcripts that peak in immature tricellular pollen (TCP). In addition, AtMIKC* complexes also repress *AGL30 *and *AGL65 *in a negative feedback loop, whereas AGL18 acts to fine tune the expression level of *AGL29*. For most genes displayed in this scheme, we confirmed the differential expression in wild-type and triple mutant pollen by reverse transcription polymerase chain reaction (Additional data file 5). In this scheme only *MYB97 *and *WRKY34 *are potential direct targets of the AtMIKC* complexes, as suggested by *in silico *analysis of their putative regulatory sequences (Additional data file 2). BCP, bicellular pollen; MPG, mature pollen grains; UNM, unicellular microspores.

Describing the complexity of a differentiation process in detailed steps is an enormous challenge. Here, we present the first analysis of a regulatory network that controls transcriptome dynamics during reproductive cell differentiation in plants. We identified AtMIKC* MADS transcription factor complexes as important regulators of the transcriptional changes that occur during pollen maturation (Figure 1). We also initiated the analysis of the regulatory network downstream of AtMIKC* complexes by identifying two non-MIKC* MADS proteins (AGL18 and AGL29) as regulators of subsets of the AtMIKC*-regulated genes. Figure 4 illustrates how the sequential action of mutually interacting transcription factors directs transcriptome dynamics during pollen maturation. The AtMIKC* complexes repress immature pollen-specific transcription factor genes such as *WRKY34*, and activate mature pollen-specific transcription factors such as *AGL18 *and *AGL29*. The proteins encoded by these two MADS box genes, in turn, predominantly repress transcripts that peak in immature tricellular pollen. In addition, the AtMIKC* complexes also repress *AGL30 *and *AGL65 *in a negative feedback loop, whereas AGL18 acts to fine tune the expression level of *AGL29*. Interestingly, *AGL18*-like genes have thus far only been reported in the Brassicaceae [45], suggesting that the incorporation of AGL18 into the AtMIKC* network in pollen is a relatively recent event.

Although this model covers only part of the much larger transcription factor network that controls pollen maturation, it represents a unique first view of plant cell differentiation in terms of a transcriptomics network. It remains an interesting challenge to investigate further the functions of the other components in this regulatory network. Virtually all other transcription factors in pollen still await thorough functional characterization, and our experiments indicate that at least 45 of these play a role in the AtMIKC* network (Additional data file 2). Our approach demonstrates that reverse genetics is feasible even for multiply redundant pollen-expressed transcription factors. Our datasets can serve as a reference tool in such approaches, which will ultimately contribute to a comprehensive and dynamic perspective of this essential cellular differentiation process.

## Materials and methods

### Plant growth conditions and pollen collection

Wild-type *A. thaliana *Col-0 plants were grown under standard greenhouse conditions, with temperature controlled at 22°C and 16 hours of light at around 120 μmol/m^2 ^per second, together with the various insertion mutants obtained from the Salk collection [46]: *agl65 *([TAIR:At1g18750]: SALK_009651), *agl66 *([TAIR:At1g77980]: SALK_072108), *agl94 *([TAIR:At1g69540]: SALK_016078), *agl104 *([TAIR:At1g22130]: SALK_098698), *agl18-2 *([TAIR:At3g57390]: SALK_144022), and *agl29 *([TAIR:At2g34440]: SALK_067236). The *agl65*, *agl66*, *agl94*, and *agl104 *alleles were also used in our previous study [28]. For practical reasons the different genotypes were grown, harvested, and processed in four separate batches, each time together with WT control plants. The first batch contained *agl66 *and *agl104*; the second *agl65*, *agl94*, and *agl65*/*66*; the third *agl66*/*104 *and *agl18-2*; and the fourth *agl65*/*66*/*104 *and *agl29*. The different batches are colour coded in the microarray overview file (Additional data file 2). For each genotype two batches of 120 plants each were grown (and three batches for *agl65*/*66*/*104 *and the WT control grown in parallel). At a fixed time in the morning, mature pollen grains were harvested from open flowers by shaking in 0.3 M mannitol, as described by Honys and Twell [6]. For total RNA isolation with the RNeasy kit (Qiagen, Hilden, Germany), freshly harvested pollen was ground with quartz sand and RNA was stored at -80°C. RT-PCR procedures are described in Additional data file 5.

### Microarray hybridization and data analysis

RNA quality was checked using a Bioanalyzer 2100 (Agilent, Palo Alto, CA, USA). Preparation of cDNA from total RNA and hybridization to ATH1 Arabidopsis Genome Arrays (Affymetrix Inc.) was performed by the Integrated Functional Genomic service unit of the Interdisciplinary Center for Clinical Research (IZKF) in Münster (Germany), in accordance with the standard manufacturer's protocol (Affymetrix GeneChip technical analysis manual). The resulting data files were normalized (scaled to a mean of 500) and analyzed with MAS5.0 software (Affymetrix), and further processed with Microsoft Excel and Access. Only genes with consistent present calls in the three WT and/or triple mutant replicates (according to MAS5.0) were considered in our analyses. We further used Cyber-T software for statistical analysis of the datasets [32], with three beta-fit iterations, and computed PPDE values based on log-transformed *P *values, with sliding window size 101 and a rather stringent confidence value of 6. When comparing WT and triple mutant samples, we only retained genes with a PPDE(p) value of at least 0.95, corresponding to a false-positive rate of 5% or less. The *P *value of all selected genes was smaller than 0.0008. PPDE values could not be obtained for the *agl66*, *agl18*, and *agl29 *samples, probably because too few genes were significantly affected in these genotypes. In these cases we then used a Bayesian log-transformed *P *value cut-off of 0.005 to identify genes that were significantly affected. When comparing single and double mutant datasets with their corresponding WT controls, we used a PPDE(p) cut-off of 0.90 to compensate for the fact that they were processed in different experiments than the triple mutant samples. For the graph in Figure 2b we used the FIRe macro [38], and for the Figure in Additional data file 3 we used MapMan [33], after preparation of the datasets using Robin software [47].

*In silico *analysis of AtMIKC* binding sites in the promoters, 5'-untranslated regions, and introns of all AtMIKC*-controlled genes was performed as described previously [28], using the bulk download tool of TAIR [48] and the binding site information experimentally obtained *in vitro *[28]. Sample purity (Additional data file 2) was assessed using the Biomarker tool in Genevestigator [49]. The expression of each putative target gene was examined in the dataset of Honys and Twell [11], which was downloaded from the *Genome Biology *website, and the pollen and/or stamen expression of putative target genes that were not expressed in the Honys and Twell dataset (see Additional data file 2) was further verified in the following three AtGenExpress samples: ATGE_36 (stamen stage 12), ATGE_43 (stamen stage 15), and ATGE_73 (mature pollen) [50].

All microarray data, with Minimum Information About a Microarray Experiment documentation, have been submitted to ArrayExpress [51], and can be found under accession number E-MEXP-1138.

### Test crosses and genotyping

For test crosses, unopened WT flowers were emasculated and hand-pollinated with pollen from plants segregating one of the AtMIKC* mutant alleles, either in a single or double mutant background (for assessment of the mutant male gametes), and reciprocal crosses were performed to assess the mutant female gametes. Progeny was genotyped for presence of the T-DNA insertions using the LBa1 primer from Salk (5'-TGGTTCACGTAGTGGGCCATCG-3') and a locus-specific primer.

### Pollen phenotyping

Light and epifluorescence microscopy of DAPI-stained pollen, including image capture and processing, were performed as described previously [52]. Viability staining of mature pollen with fluorescein diacetate and *in vitro *pollen germination assays were carried out as described previously [24,28,53].

*In vivo *pollen tube growth in pistils was visualized with decolorized aniline blue, as described previously [54]. Pistils were fixed 2.5 hours after pollination. Pollen dehydration was tested with HPTS (Sigma, St-Louis, MO, USA), in accordance with the method reported by Johnson and McCormick [31].

For surface analysis, mature pollen grains from open flowers of WT and triple mutants were mounted onto aluminium stubs with double-sided adhesive tape and gold coated with a sputter coater (Polaron SC7640; Quorum Technologies, Ringmer, UK). Coated samples were observed, and digital images captured, using a Hitachi S3000H scanning electron microscope. Fixation, embedding in Spurr's resin, and ultrastructural analysis of pollen in mature nondehiscent anthers were carried out essentially as described previously [55]. Observations were made, and digital images captured, using a JEOL 1200 transmission electron microscope (JEOL UK Ltd, Welwyn Garden City, UK). For staining of cell wall components, Spurr's resin embedded sections of mature anthers 1 mm thick were treated as follows: callose (0.03% [weight/vol] decolorized aniline blue [Sigma] in 0.1 mol/l K_3_PO_4_; pH11), cellulose (0.01% [weight/vol] calcofluor [Fluorescent Brightener 28; Sigma] in 0.1 mol/l Tris-HCl buffer; pH 9.0), and pectin (0.01% [weight/vol] Ruthenium red [Sigma] or 1% [weight/vol] Alcian blue 8GX [Sigma] in 3% acetic acid). Sections were stained for 15 minutes at room temperature (aniline blue, calcofluor), or 30 minutes at 50°C (Ruthenium red, Alcian blue), rinsed in water, and examined with light (pectin stains) or fluorescence microscopy (aniline blue: Excitation λ 450 to 490 nm and Emission λ >500 to 545 nm; calcofluor: Excitation λ 340 to 380 nm and Emission λ 435 to 485 nm). Fluorescence images were captured with a Hamamatsu Orca ER camera using Open Lab software (Improvision, Coventry, UK), and color images were captured with a Nikon D100 camera on a Nikon TE-2000E inverted microscope.

### Yeast-three-hybrid analysis

For the yeast-three-hybrid assay the previously identified AtMIKC* dimers [28] were reconstituted in yeast strain PJ69-4A (matA [56]). For this purpose, the gene encoding one of the two dimerization partners was cloned into vector pTFT1 [41] and co-transformed into the indicated yeast strain with the pADGAL4-vector expressing its dimerization partner [57]. Subsequently, the obtained yeast clones were combined by mating with yeast PJ69-4α (matα) clones that contain a pBDGAL4 vector harbouring one of the AtMIKC* MADS box genes, *AGL18 *or *AGL29*, as insert, respectively. Mating was performed as described by de Folter and colleagues [53], and yeast cells containing all three plasmids were selected on medium lacking leucine, tryptophan, and adenine. Afterward, these yeast cells were re-suspended in 100 μl water and spotted in 5 μl droplets onto selective medium lacking leucine/tryptophan/adenine and histidine, and supplemented with 1, 5, or 10 mmol/l 3-amino-1,2,4-triazole. These plates were incubated at room temperature for 5 days, followed by scoring of yeast growth, in order to identify ternary complex formation. To confirm the identified protein interactions a LacZ screen was performed on the same clones, as described previously [58]. Combinations that were positive on at least two selective media were scored as true interaction events.

## Abbreviations

BCP, bicellular pollen; DAPI, 4',6-diamidino-2-phenylindole; HPTS, 8-hydroxypyrene-1,3,6-trisulfonic acid; IP, immature pollen; MP, mature pollen; MPG, mature pollen grains; PPDE, posterior probability of differential expression; RT-PCR, reverse transcription polymerase chain reaction; TCP, tricellular pollen; T-DNA, transferred DNA; UDP, uridinediphosphate; UNM, unicellular microspores; WT, wild-type.

## Authors' contributions

WV conceived of the experiments, identified the insertion mutants, and constructed the double and triple mutant combinations; he carried out pollen germination assays and test crosses, performed all pollen transcript profiling experiments, analyzed all resulting data, was responsible for *in silico *promoter analysis, and wrote the manuscript. DT conceived of the experiments and performed all morphologic analyses of mutant and WT pollen, as well as pollen germination assays; he contributed to scientific discussion and writing of the manuscript. SdF and RI carried out the yeast-three-hybrid analysis and contributed to scientific discussion. HS contributed to scientific discussion. TM conceived of the experiments and contributed to scientific discussion.

## Additional data files

The following additional data are available with the online version of this paper. Additional data file 1 shows phenotypic characterization of triple mutant pollen. Additional data file 2 provides an overview of microarray datasets and data analyses. Additional data file 3 illustrates how AtMIKC* complexes regulate cell wall component synthesis in pollen. Additional data file 4 shows putative AtMIKC* target gene categories. Additional data file 5 provides RT-PCR confirmation of putative AtMIKC* target genes. Additional data file 6 shows that AtMIKC* proteins form higher order complexes in yeast.

## Supplementary Material

Additional data file 1 As described in Materials and methods, we examined various functional and morphological aspects of WT and *agl65/66/104 *triple mutant pollen grains. Apart from (a) a nearly complete block in *in vitro *pollen germination, triple mutant pollen was indistinguishable from the WT, with respect to (b) *in vivo *germination and pollen tube growth, (c) viability and membrane integrity, (d) nuclear number and organization, (e) surface appearance, (f) ultrastructure, (g) cellulose, (h) callose, and (i,j) pectin. Click here for file

Additional data file 2 This Excel file contains relevant information about the genes consistently affected in triple mutant pollen, including all related information we used in figures and calculations. Separate legends are included in each worksheet. Click here for file

Additional data file 3 In this presentation based on MapMan [33], part of the synthesis pathway for cell wall components is shown. Blue boxes indicate that a gene responsible for a reaction is upregulated in triple mutant pollen, and red boxes indicate downregulated genes. For each gene the expression profile throughout pollen development is added (based on Honys and Twell [11]). Almost without exception immature-pollen genes are upregulated in triple mutant pollen, while the few mature pollen genes in this pathway are downregulated. Click here for file

Additional data file 4 Based on the general trend of their expression in the different AtMIKC* single and double mutants - which reflects the impact of loss of the individual AtMIKC* complexes on their expression level - we defined five categories of AtMIKC*-regulated genes. Click here for file

Additional data file 5 For a selection of the genes affected in *agl65/66/104 *triple mutant pollen (identified by transcriptome analysis) we verified the differential expression in WT and triple mutant pollen by RT-PCR, using RNA from independently harvested pollen samples. Click here for file

Additional data file 6 In a yeast-three-hybrid experiment the previously identified AtMIKC* heterodimeric complexes [28] were tested for ternary interaction with the individual AtMIKC* proteins, and with AGL18 and AGL29. At least nine AtMIKC* higher-order complexes could be reliably detected in yeast. AGL18 and AGL29 did not interact with AtMIKC* proteins. Click here for file
