# Supplementary material for: MADS-complexes regulate transcriptome dynamics during pollen maturation
Source: Genome Biol. 2007 Nov 22;8(11):R249. doi: 10.1186/gb-2007-8-11-r249 (PMC2258202; doi:10.1186/gb-2007-8-11-r249)
Supplement: Additional data file 1 — As described in Materials and methods, we examined various functional and morphological aspects of WT and agl65/66/104 triple mutant pollen grains. Apart from (a) a nearly complete block in in vitro pollen germination, triple mutant pollen was indistinguishable from the WT, with respect to (b) in vivo germination and pollen tube growth, (c) viability and membrane integrity, (d) nuclear number and organization, (e) surface appearance, (f) ultrastructure, (g) cellulose, (h) callose, and (i,j) pectin. [file gb-2007-8-11-r249-S1.pdf]

<u>Additional data file 1</u>: **Phenotypic characterization of triple mutant pollen**

a. *In vitro* pollen germination

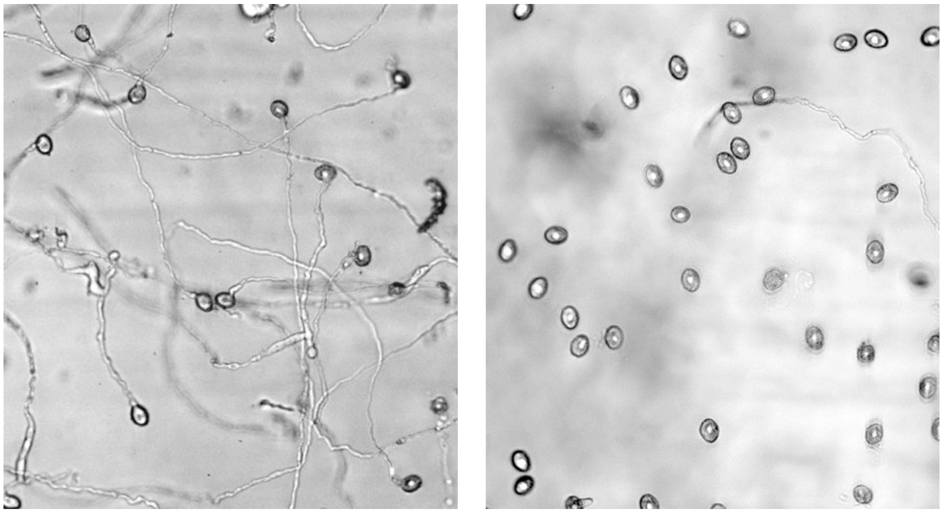

Wt Col-0 *agl65/66/104*

b. *In vivo* pollen tube germination and growth: aniline blue

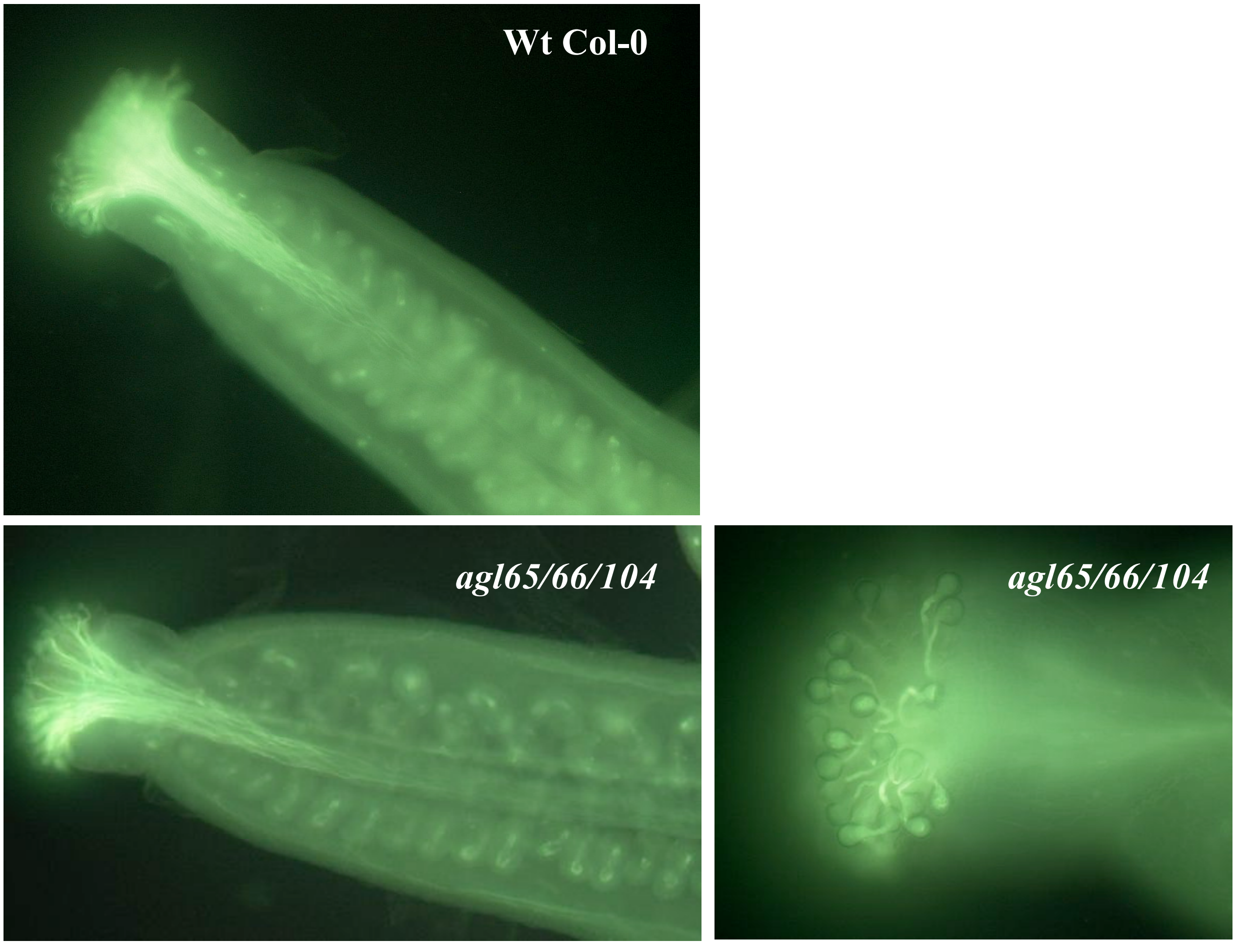

c. Staining for viability and membrane integrity: fluoresceine diacetate

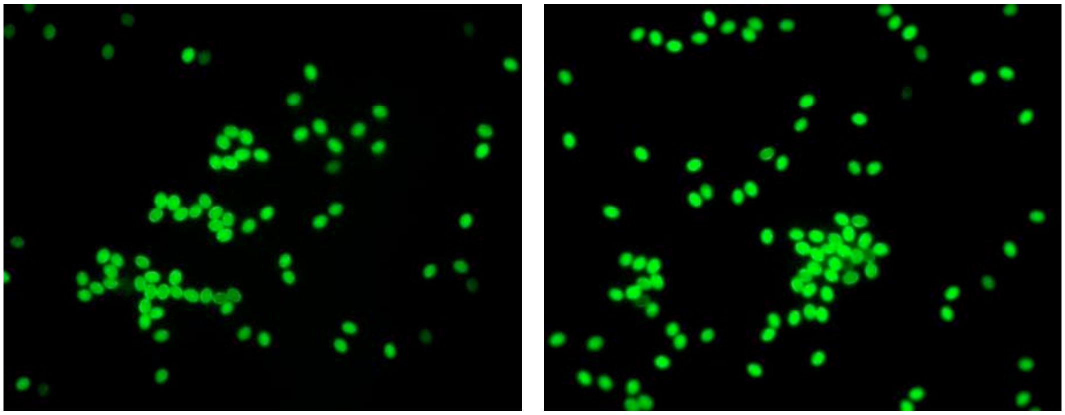

Wt Col-0 *agl65/66/104*

d. Nuclear staining: DAPI

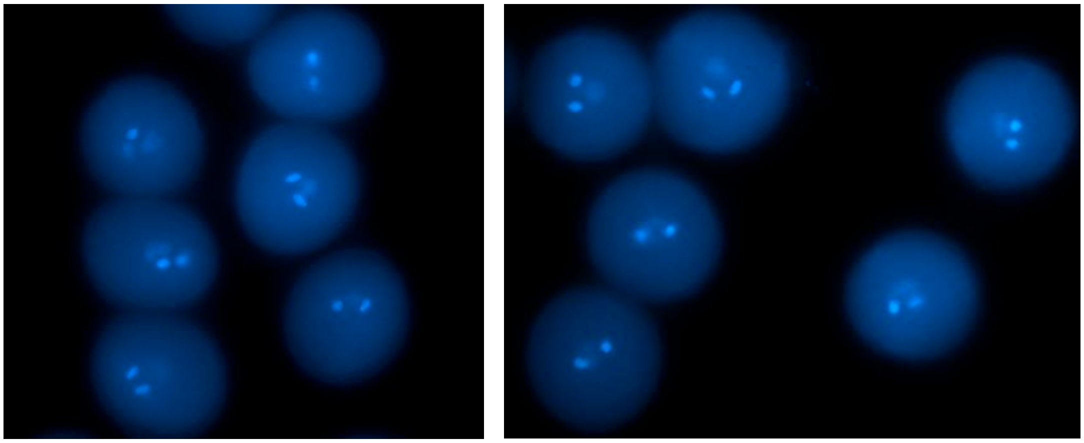

Wt Col-0 *agl65/66/104*

e. Pollen surface: scanning electron microscopy

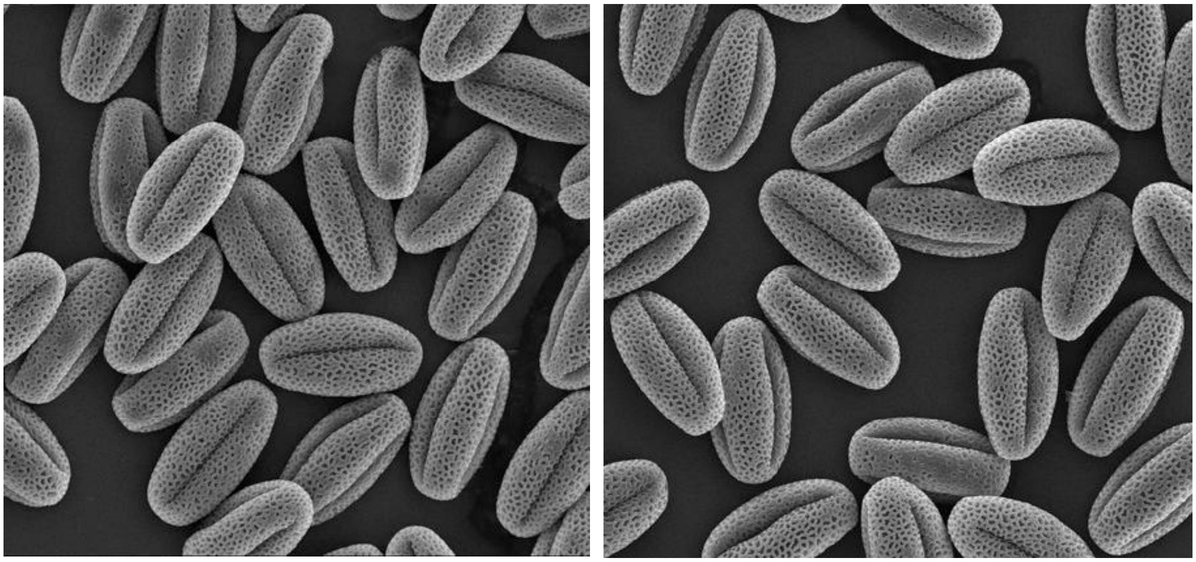

Wt Col-0 *agl65/66/104*

f. Pollen ultrastructure: transmission electron microscopy

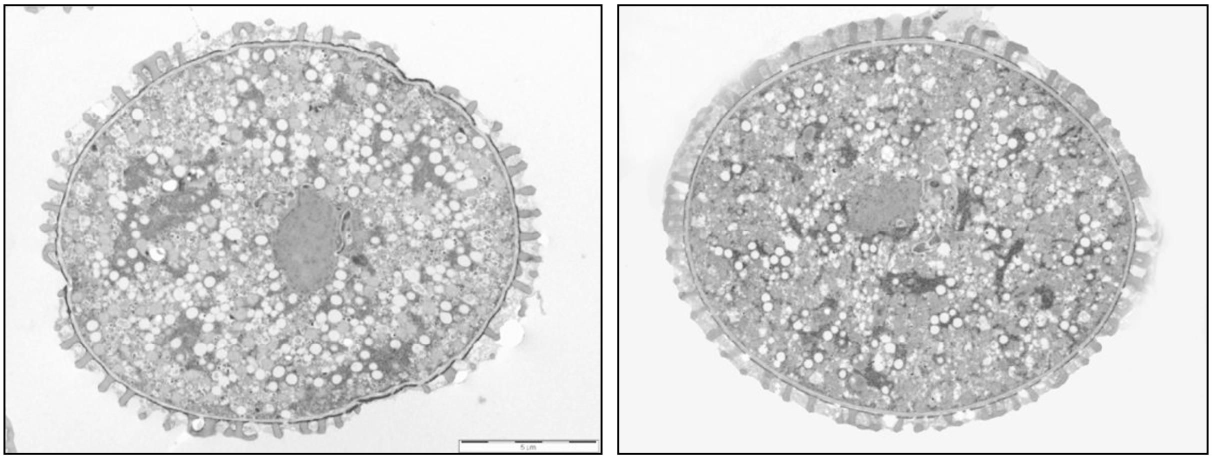

Wt Col-0 *agl65/66/104*

g. Staining for cellulose: calcofluor

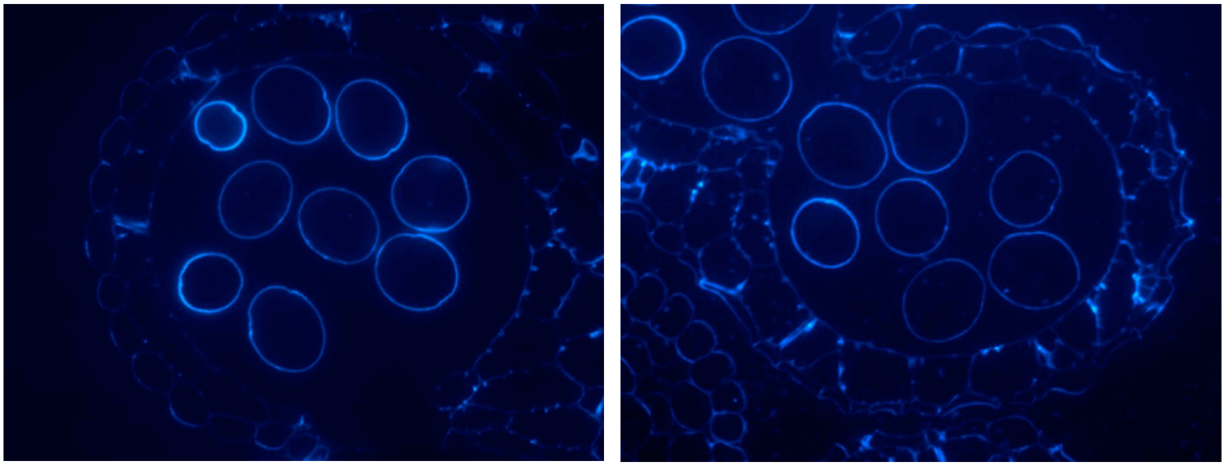

Wt Col-0 *agl65/66/104*

h. Staining for callose: aniline blue

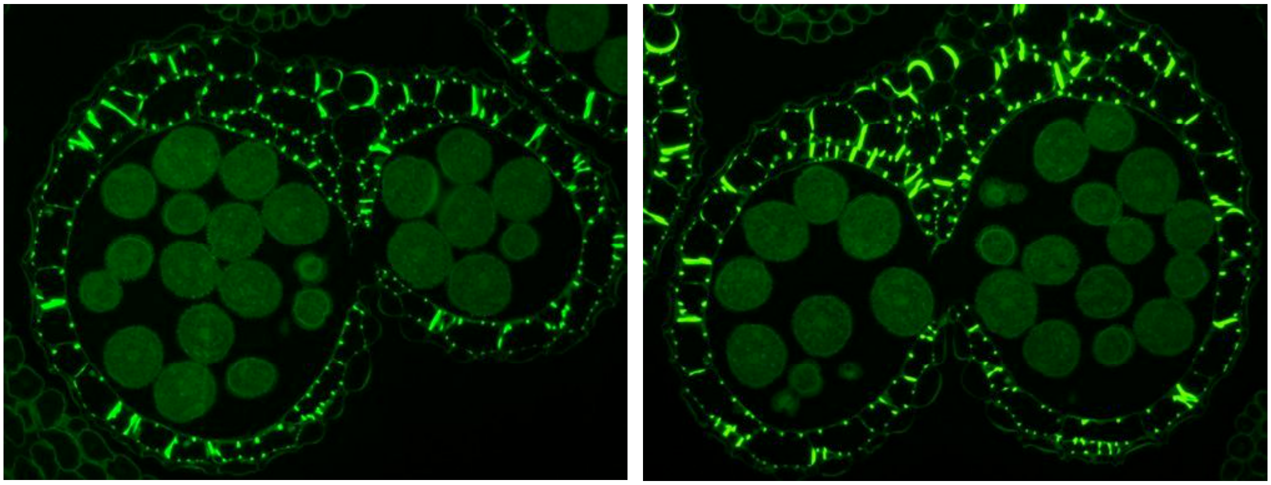

Wt Col-0 *agl65/66/104*

i. Staining for pectins: ruthenium red

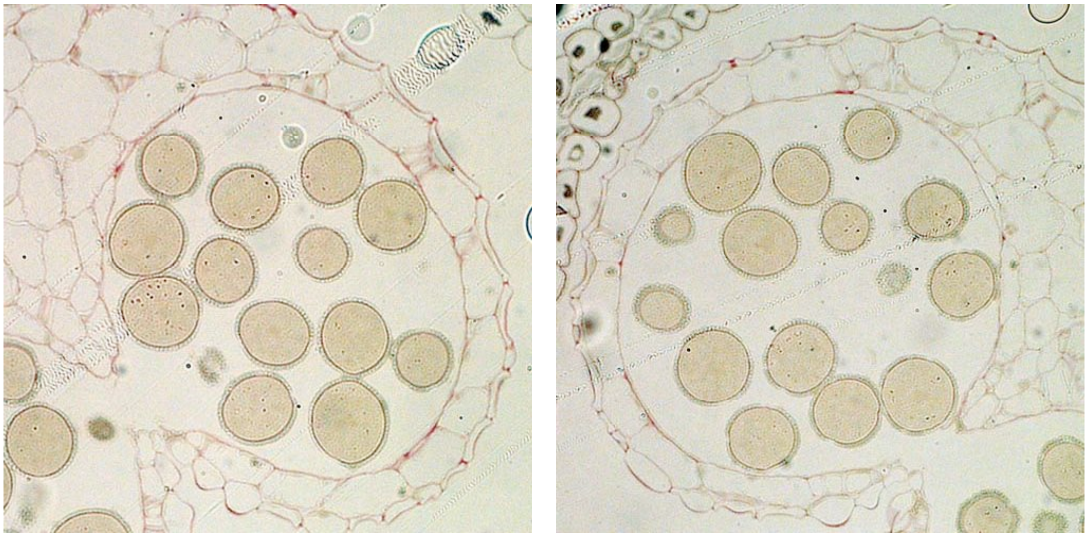

Wt Col-0 *agl65/66/104*

j. Staining for pectins: alcian blue

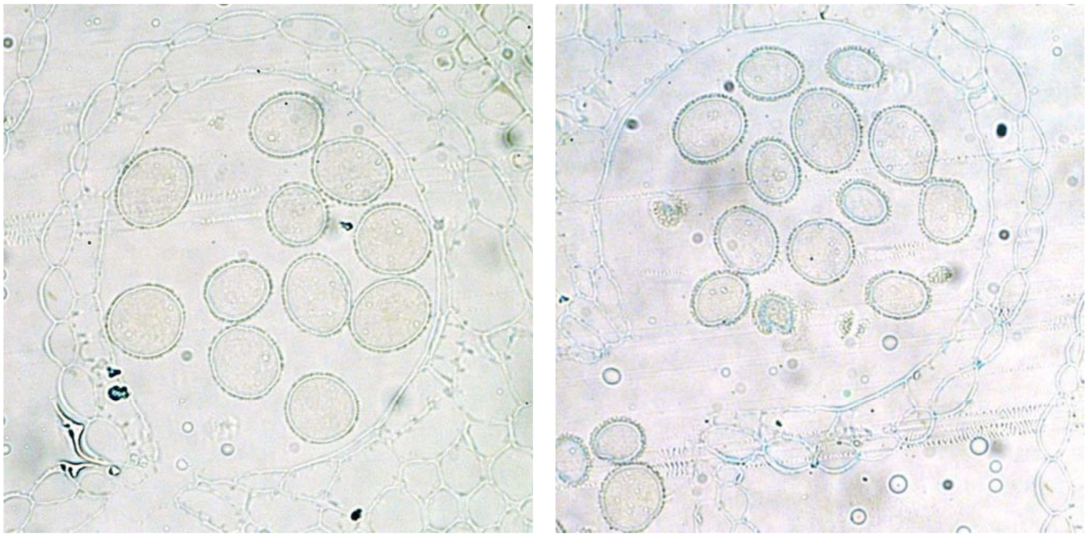

Wt Col-0 *agl65/66/104*
