# Supplementary figures and images for: MADS-complexes regulate transcriptome dynamics during pollen maturation
Source: Genome Biol. 2007 Nov 22;8(11):R249. doi: 10.1186/gb-2007-8-11-r249 (PMC2258202; doi:10.1186/gb-2007-8-11-r249)

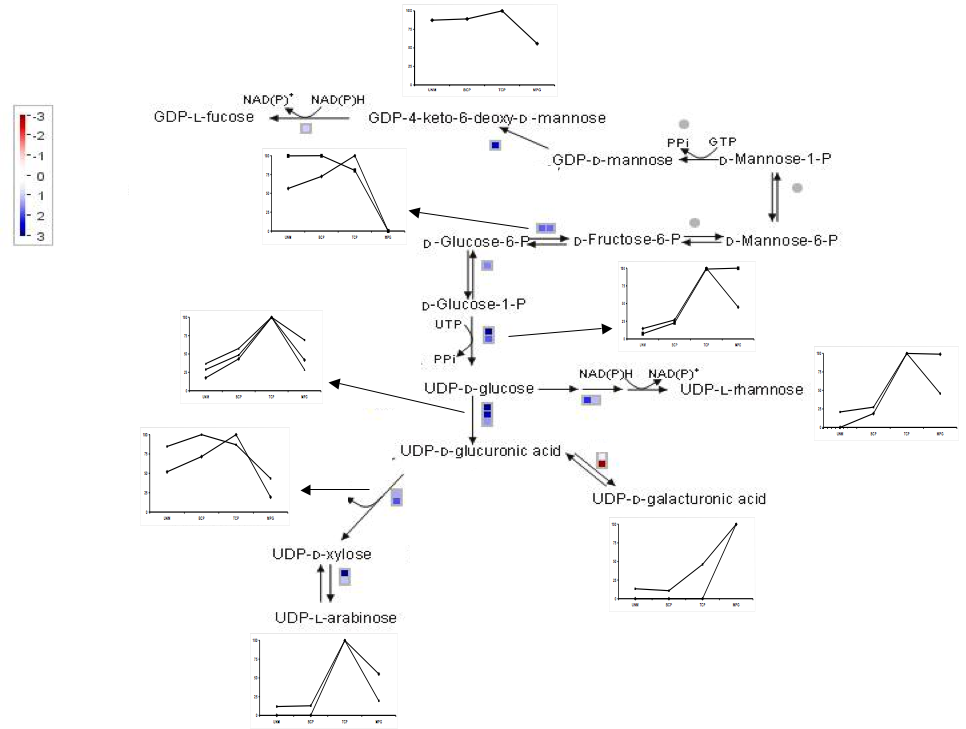

Supplement: Additional data file 3 — In this presentation based on MapMan [33], part of the synthesis pathway for cell wall components is shown. Blue boxes indicate that a gene responsible for a reaction is upregulated in triple mutant pollen, and red boxes indicate downregulated genes. For each gene the expression profile throughout pollen development is added (based on Honys and Twell [11]). Almost without exception immature-pollen genes are upregulated in triple mutant pollen, while the few mature pollen genes in this pathway are downregulated. [file gb-2007-8-11-r249-S3.tiff]
