# Supplementary material for: MADS-complexes regulate transcriptome dynamics during pollen maturation
Source: Genome Biol. 2007 Nov 22;8(11):R249. doi: 10.1186/gb-2007-8-11-r249 (PMC2258202; doi:10.1186/gb-2007-8-11-r249)
Supplement: Additional data file 4 — Based on the general trend of their expression in the different AtMIKC* single and double mutants - which reflects the impact of loss of the individual AtMIKC* complexes on their expression level - we defined five categories of AtMIKC*-regulated genes. [file gb-2007-8-11-r249-S4.pdf]

Additional data file 4: Categories of AtMIKC*-regulated genes

To get a deeper insight into the redundancy between the AtMIKC* complexes, we examined the behaviour of the AtMIKC*-controlled genes in the various single and double mutants more closely, also taking the extent of changes in expression level into account. We used a one-sided Student's t-test (with 90% confidence interval as cut-off, see Additional data file 2) to identify significant differences between the expression levels in the Wt, single, double and triple mutants (see materials and methods). Based on the general trend of their expression in these different mutants – which reflected the impact of loss of the individual AtMIKC* complexes on their expression level – we defined five categories of AtMIKC*-regulated genes. In the accompanying graphs two examples are given for each category, including their expression level in Wt, *agl65*, *agl65/66*, *agl66/104* and *agl65/66/104* mutant pollen, relative to the Wt level. All data are derived from microarray experiments [Additional data file 2]. Arrows point at genotypes in which the gene is significantly affected relative to the genotype(s) to the left of it. The total number of genes we identified for each category is mentioned on the right. All calculations and gene lists can be found in Additional data file 2.

The presence (green), reduced abundance (orange) and absence (red) of each AtMIKC* complex in the genotypes included in this analysis is shown in the following overview table.

		Mutant background			
		agl65	*agl65/66*	*agl66/104*	*triple*
COMPLEX	AGL30/66	PRESENT	ABSENT	ABSENT	ABSENT
	AGL65/66	ABSENT	ABSENT	ABSENT	ABSENT
	AGL94/66	PRESENT	ABSENT	ABSENT	ABSENT
	AGL30/104	PRESENT	PRESENT	REDUCED	REDUCED
	AGL65/104	ABSENT	ABSENT	REDUCED	ABSENT

PRESENT
REDUCED
ABSENT

Category 1: Genes redundantly regulated by AGL65-complexes

Genes that are exclusively and redundantly regulated by the AGL65-complexes should ideally be affected to the same extent in all mutants with interrupted *AGL65* locus, *i.e.* in *agl65*, *agl65/66* and *agl65/66/104*. We observed this tendency for 21 genes. Functional loss of additional AtMIKC* complexes did not significantly alter their expression level further.

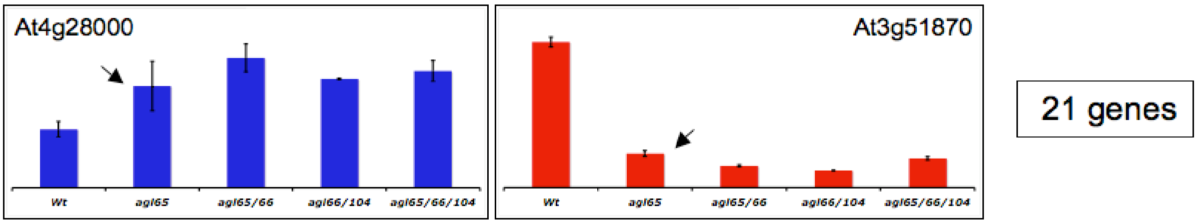

Category 2: Genes additively regulated by AGL30- and AGL65-complexes

Genes additively regulated by the AGL65- and AGL30-complexes should be affected in the *agl65* mutant, but the loss of additional complexes should further enhance this tendency in the triple mutant. A total of 60 genes met these requirements.

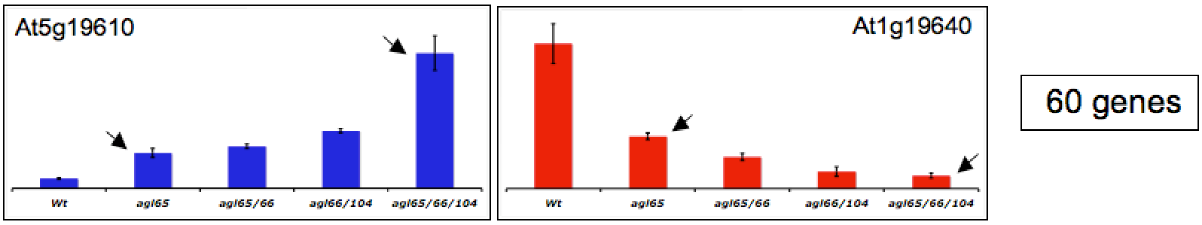

Category 3: Genes redundantly regulated by AGL30-complexes

AtMIKC*-controlled genes redundantly regulated by the AGL30-complexes alone should be unaffected by the functional loss of only the AGL65-complexes (in the *agl65* mutant), and by the additional functional loss of just one of the AGL30-complexes (in *agl65/66*). This category of genes would thus only be affected in the *agl66/104* and *agl65/66/104* mutants. In addition, they should be affected to a comparable extent in both these mutants, because the triple mutant only differs from *agl66/104* in the additional functional loss of the AGL65/104 complex, which should not affect the expression of these genes. A total of 657 genes met these criteria.

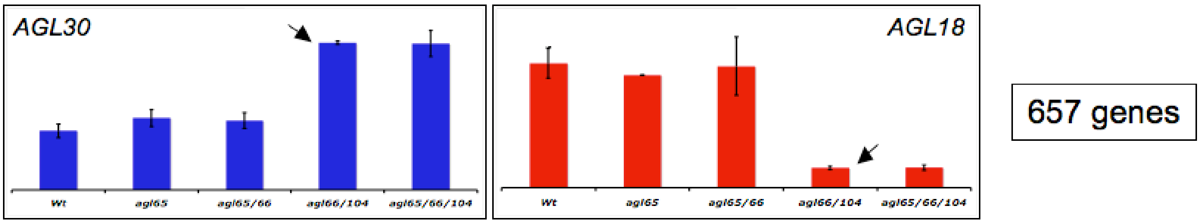

Category 4: Genes redundantly regulated by AGL30- and AGL65-complexes

A fourth category – containing 133 genes – showed a similar behaviour, but their expression did change significantly in triple mutant pollen, relative to *agl66/104*. These genes are likely redundantly regulated by the AGL30- and AGL65-complexes.

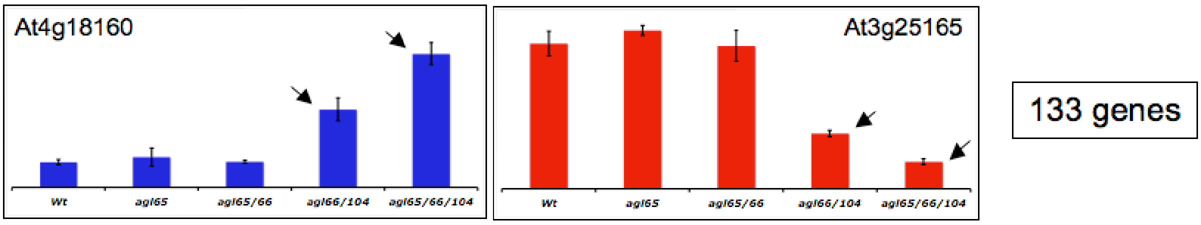

Category 5: Genes redundantly regulated by AGL30- and AGL65-complexes, with lower threshold level

A last category of genes was affected in the *agl65/66/104* triple mutant, but not in any of the other mutants we investigated. We found 85 genes that behaved according to this pattern. Like category 4, these are also redundantly regulated by the AGL30- and AGL65-complexes, but the difference is that they likely require lower levels of AtMIKC* complex. The functional presence of one AtMIKC* complex at normal abundance (in *agl65/66*) or two complexes at low abundance (in *agl66/104*) is sufficient to ensure their proper transcriptional regulation. Their expression level is only changed when AGL30/104 levels are reduced in addition to the functional loss of all other AtMIKC* complexes (in *agl65/66/104*).

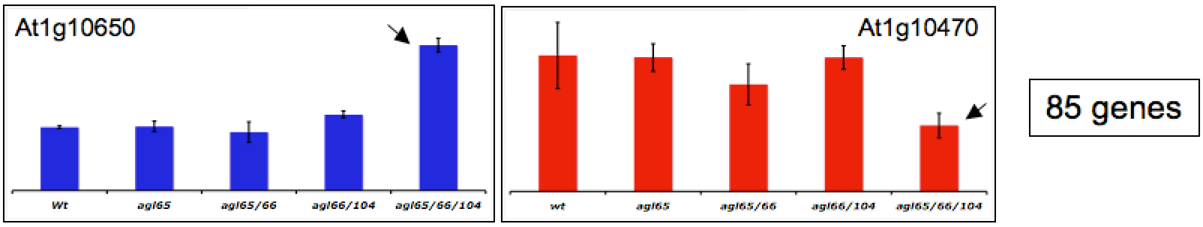

Conclusion

Only 21 genes seem to be exclusively regulated by the AGL65-complexes (category 1), while the AGL30-complexes regulate at least 657 genes independently from the AGL65-complexes (category 3). Functional overlap is quite considerable between AGL30- and AGL65-complexes: together they redundantly regulate at least 218 genes (categories 4 and 5 together). Our analysis also indicates that some downstream-regulated genes require higher

levels of AtMIKC* complex for their proper regulation than others do. This difference is illustrated by categories 4 and 5.
