# Supplementary material for: MADS-complexes regulate transcriptome dynamics during pollen maturation
Source: Genome Biol. 2007 Nov 22;8(11):R249. doi: 10.1186/gb-2007-8-11-r249 (PMC2258202; doi:10.1186/gb-2007-8-11-r249)
Supplement: Additional data file 5 — For a selection of the genes affected in agl65/66/104 triple mutant pollen (identified by transcriptome analysis) we verified the differential expression in WT and triple mutant pollen by RT-PCR, using RNA from independently harvested pollen samples. [file gb-2007-8-11-r249-S5.pdf]

Additional data file 5: RT-PCR confirmation of putative AtMIKC* target genes

Transcript profiling indicated that 1353 genes are affected in *agl65/66/104* triple mutant pollen [Additional data file 2]. For a selection of these genes we verified their differential expression in Wt and triple mutant pollen, by reverse transcriptase PCR, using RNA from independently harvested pollen samples (Wt and triple mutant). The selection includes two sporophytic genes that appear to be repressed in pollen by the AtMIKC* complexes (*MS2* and *TIR1*), the transcription factors included in figure 4 (*AGL18*, *AGL29*, *MYB97*, *WRKY34*, *CCA1* and *SCL8*), and the gene encoding the key enzyme for abscisic acid synthesis in pollen (*NCED6*). For each gene the fold-change relative to Wt pollen is mentioned, as determined by microarray. PCR conditions are described below.

Gene name	AGI number	Wt triple	Fold change
NCED6	*At3g24220*	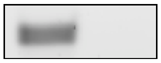	- 24.2
AGL18	*At3g57390*	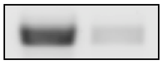	- 6.3
AGL29	*At2g34440*	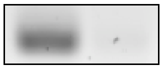	- 5.7
MYB97	*At4g26930*	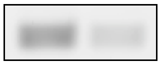	- 4.2
ARR2	*At4g16110*	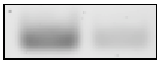	- 3.2
ZTL	*At5g57360*	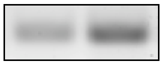	2.7
WRKY34	*At4g26440*	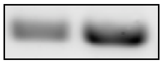	3.8
SCL8	*At5g52510*	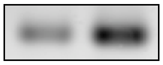	9.9
CCA1	*At2g46830*	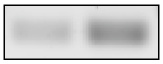	17.0
TIR1	*At3g62980*	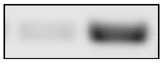	30.4
MS2	*At3g11980*	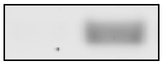	62.2
	18S rRNA	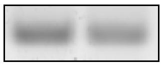	

Materials and methods for RT-PCR

Total RNA was isolated from mature pollen as described [28], which had been harvested from wild-type and *agl65/66/104* triple mutant plants grown in a greenhouse, with temperature controlled at 22ºC and 16 hours of light at around 120 µmol m^{-2} s^{-1}. For cDNA synthesis (with 5 µg of total RNA) an oligo-d(T) primer was used, together with the Superscript II RNase H-Reverse Transcriptase kit (Invitrogen, Carlsbad, CA). Primers were designed in the 3' region of the open reading frame, and wherever possible spanned an intron. Reverse-transcriptase PCR was performed using 150 ng cDNA and gene-specific primers. As a control, 18S ribosomal RNA was amplified, using the QuantumRNA primer-competimer approach from Ambion (Austin, TX), with a 2:8 ratio of primer-competimer and 35 PCR-cycles. The number of PCR-cycles was optimized for each gene: 25 cycles for *JMT*, *MYB97*, *AGL18*, *ARR2*, *MS2* and *ZTL*; 30 cycles for *WRKY34*, *NCED6*, *TIR1*, *SCL8* and *CCA1*; 35 cycles for *AGL29*.
