# Supplementary material for: MADS-complexes regulate transcriptome dynamics during pollen maturation
Source: Genome Biol. 2007 Nov 22;8(11):R249. doi: 10.1186/gb-2007-8-11-r249 (PMC2258202; doi:10.1186/gb-2007-8-11-r249)
Supplement: Additional data file 6 — In a yeast-three-hybrid experiment the previously identified AtMIKC* heterodimeric complexes [28] were tested for ternary interaction with the individual AtMIKC* proteins, and with AGL18 and AGL29. At least nine AtMIKC* higher-order complexes could be reliably detected in yeast. AGL18 and AGL29 did not interact with AtMIKC* proteins. [file gb-2007-8-11-r249-S6.pdf]

Additional data file 6: Yeast-three-hybrid analysis

Dimers	AD	TFT	BD						
			AGL65	AGL104	AGL29	AGL18	AGL30	AGL94	AGL66
1	AGL30	AGL66	A/B/C/D	A/B/C/D	-	-	A/B	-	A/B/C/D
2	AGL66	AGL30	A	A	-	-	A	-	A/B/C/D
3	AGL30	AGL104	A/B/C/D	A/B/C/D	-	-	A/B/C/D	-	A/B/C/D
4	AGL104	AGL30	A/B/C/D	A	-	-	A/B/C/D	-	A/B/C/D
5	AGL65	AGL66	A/B/C/D	A/B/C/D	-	-	-	-	A/B/C/D
6	AGL66	AGL65	A/B/C/D	A/B/C/D	-	-	A/B/C	-	A/B/C/D
7	AGL65	AGL104	ND	ND	ND	ND	ND	ND	ND
8	AGL104	AGL65	A/B/C/D	A/B/C/D	-	-	A/B/C/D	-	A/B/C/D

Yeast growth on respective media:

A	**-LTAH**
B	**-LTAH + 1 mM 3AT**
C	**-LTAH + 5 mM 3AT**
D	**-LTAH + 10 mM 3AT**
-	**no growth**
ND	**not done**

Observed higher-order complexes*:

**1**	AGL30	AGL66	AGL65
**2**	AGL30	AGL104	AGL65
**3**	AGL30	AGL66	AGL30
**4**	AGL30	AGL104	AGL30
**5**	AGL65	AGL66	AGL65
**6**	AGL66	AGL30	AGL104
**7**	AGL66	AGL65	AGL104
**8**	AGL104	AGL30	AGL104
**9**	AGL104	AGL65	AGL104

*Scoring criteria:
All combinations that were positive on at least two selective media and by the LacZ screen were scored as true interaction events. Furthermore, some interaction events could not be scored as positive due to dimer formation (Y2H: see [28]), or autoactivation (AGL66).
